# This is the End: The Role of Death in the Narrative Structure of Popular Movies

**DOI:** 10.1177/00302228241280311

**Published:** 2024-08-29

**Authors:** Kobie van Krieken, Enny Das

**Affiliations:** Centre for Language Studies, 6029 Radboud University, Nijmegen, The Netherlands

**Keywords:** death, movie, narrative structure, eudaimonic entertainment

## Abstract

A set of sixty popular movies is analyzed both quantitatively and qualitatively on the role of death in the narrative structure and the portrayal of death. Results of the quantitative analysis show that death events tend to be story-terminating, which implies that death is typically depicted as meaningful in relation to the past. The qualitative thematic analysis reveals that death is also depicted as meaningful in that it can lead to growth, unification and salvation. Furthermore, most movies include explicit death portrayals, thereby inviting viewers to closely approach death and to simulate both their own mortality and the mortality of their loved ones. Finally, it was found that most movie deaths involve violent attacks, indicating that movies tend to paint an unrealistic view of how people die. These findings advance our knowledge of how movies might help people to understand the meaning of death and to cope with existential fears.

## Introduction

Death is a central topic in many movies. Characters die in war (e.g., John Miller in *Saving Private Ryan*), succumb to illnesses (e.g., Gus in *The Fault in our Stars*), are murdered (e.g., Marion Crane in *Psycho)*, drown (e.g., Billy Tyne in *The Perfect Storm*), take their own life (e.g., Neil Perry in *Dead Poets Society*) or have fatal allergic reactions (e.g., Thomas J. in *My Girl*). When watching a movie, viewers are on average confronted with a death-related scene every seven to 8 minutes (Schultz & Huet, 2001). Critical voices have argued that movies may lead to an unrealistic view of reality among viewers and contribute to unhealthy death attitudes (Fulton & Owen, 1988; see; Niemiec & Schulenberg, 2011).

However, tragic movies about death and loss may also have positive effects on viewers. In the realm of research on eudaimonic entertainment, which refers to meaningful entertainment that moves and touches the audience (Oliver & Raney, 2011; Wirth et al., 2012), studies have demonstrated the potential of tragic movies to decrease viewers’ fear of death and to stimulate them to process emotions related to the fear of losing someone (Das & te Hennepe, 2022; Rieger & Hofer, 2017). These studies suggest that movies may influence people’s outlook on and way of dealing with death. To gain a better understanding of *what* viewers may learn about death from movies, this study sets out to investigate *how* death is portrayed in popular movies. It focuses in particular on the role of death in the narrative structure of movies, because this reflects if death is understood to be meaningful in relation to the future or the past (Hagin, 2010).

The current study is grounded in the research domains of media psychology, communication studies, and death studies. In the next section, we start out by discussing previous analytical and experimental research on death in movies. We then explain how narratives function as a flight simulator by enabling audience members to virtually experience unknown, socially complex or threatening situations in a risk-free environment (e.g., Oatley, 2016). This will be followed by a discussion of the structure of narratives and research on the role of death in the narrative structure of movies, after which we will introduce our study.

### Death in Movies

Several studies have analyzed death scenes in movies with the goal to understand what viewers may learn about death from movies and how movies might affect viewers’ attitudes towards death. Schultz and Huet (2001) analyzed death scenes in 65 American movies that were nominated for an Academy Award for best picture and/or topped the list of highest grossing movies between 1980 and 1994. Results showed that these movies included a total of 857 death-related scenes, which corresponds to an average of 13 death-related scenes per movie. The majority of death-related scenes were attacks, followed by threats and risks and conversations about death. Scenes involving accidents, medical deaths, self-harm events, and funerals or burials were rare. These results lead Schultz and Huet (2001, p. 146) to conclude that “entertainment films offer a grossly-distorted view of death” which is dominated by sensation and violence.

Content analyses of Disney and Pixar films have resulted in more optimistic conclusions, by showing that these movies offer models for grieving, in particular for children: they tend to show that death is irreversible, that both good and bad people die, and that their death elicits negative emotions in their loved ones who use realistic coping styles to deal with their grief (Cox et al., 2005; Graham et al., 2018). Disney and Pixar films have therefore been argued to advance children’s understanding of death and to offer valuable opportunities to start conversations about death and grief with children (Tenzek & Nickels, 2019).

The contention that movies about death may have a positive impact on viewers has been tested in several experimental studies. These studies have shown that eudaimonic movies can serve as an anxiety buffer against the fear of death (Hofer, 2013; Rieger et al., 2015). Other studies showed that under certain circumstances, movies about loss invite viewers to experience emotions related to death and loss (Das & te Hennepe, 2022) and may decrease death avoidance (Das et al., 2019). These findings resonate with the idea that narratives function as a ‘flight simulator’ (Oatley, 2008). Like any other type of narrative, movies create imaginative worlds. Upon entering such an imaginative world, audience members mentally simulate the social scenarios occurring in it (Black et al., 2021; Boyd, 2009). This simulation can be seen as a preparation for when these scenarios might occur in real life (Van Krieken, 2018). Indeed, much like how a flight simulator improves flying skills, fictional stories might improve people’s skills to navigate social life (Oatley, 2016).

In the context of death, movies depicting death can thus be argued to offer a virtual experience in which people can safely approach and simulate death and learn to assign meaning to death (Gibson, 2001). It is yet unclear, however, what exactly viewers learn about death from watching movies and simulating death-related scenes. Counting numbers of deaths is not sufficient to arrive at a thorough understanding of the function and meaning of death in movies. This study therefore analyzes not only if and how, but also why movie characters die, by focusing on the role and meaning of death in the narrative structure of movies, and how their death is depicted.

### Death and the Narrative Structure of Movies

Narrative structure refers to the event order in a story. Every story has two structures: an underlying narrative structure (also referred to as *fabula*), which is the chronological order of the events, and a surface structure, which is the order in which the events are told or shown in the story (also referred to as *syuzhet*; Brewer & Lichtenstein, 1982; Propp, 1928). The surface structure does not have to be chronological and correspond to the underlying narrative structure. In fact, in many stories the surface structure diverges from the underlying structure, for example through the use of flashbacks and flashforwards (e.g., Cameron, 2008).

The relation between the role of death in the narrative structure of movies and the meaning of death is the focus of a framework developed by Hagin (2010). According to Hagin (2010), death can fulfill three roles in the structure of a movie. Death can be (1) story-initial, in which case it is an event that causes the subsequent events and initiates the narrative plot; (2) story-intermediate, in which case it is an event that is an effect of certain events as well as a cause of other events; or (3) story-terminating, in which case it is an effect of previous events and marks the ending of the story. Story-initial deaths are meaningful in relation to the future, meaning that they can alter or set up new goals for a character or put a character in a new social position (Hagin, 2010). Movies with a story-initial death may thus show that a person’s death, however sad and heartbreaking, may add purpose and meaning to the future lives of those left behind. Story-terminating deaths are meaningful in relation to the past, either by revealing the truth or by making an end to evil (Hagin, 2010). In the first scenario, a character’s death may for example lead to the disclosure of a secret and reveal the truth about what already has taken place. An example of the second scenario is when a vicious character is killed because he could not be stopped otherwise. Such deaths are meaningful in relation to the past because they put an end to this – often bloody and cruel – past. Finally, story-intermediate deaths are meaningful in relation to the past as well as the future.

The current study employs Hagin’s (2010) framework to analyze the role of death in the underlying narrative structure of movies:

RQ1 How is death related to the narrative structure of popular movies?

### Portrayal of Death

Additional factors may contribute to how movies assign meaning to death and may influence what viewers exactly simulate while watching death scenes, such as the role of the character who passes away. For example, Gibson (2001) argues that the main character in action movies, the hero of the story, never dies within the story’s timeframe. This would imply that viewers of death scenes in action movies, when identifying with the main character, never simulate their own death but the confrontation with another person’s death. Such movies may affect perceptions of loss and mourning, but will not affect (coping with) the most fundamental human fear of one’s own death (Greenberg et al., 1986).

The way in which death is depicted can also play a role in viewers’ simulation of death scenes. Previous analytical studies made a distinction between explicit death portrayals and implicit death portrayals (Cox et al., 2005; Graham et al., 2018). In case of an explicit death portrayal, a character’s dead body is shown. In case of an implicit death, the movie makes clear that a character is dead without showing it, for example by means of a conversation or a symbolic suggestion. Hence, viewers approach death more closely, and in a sense more realistically, if a character’s death is shown explicitly rather than implicitly.

A final relevant factor is the type of death. In their analysis of popular American movies, Schultz and Huet (2001) found a variety of death events, including attacks, medical deaths, accidents, and funerals. This implies that movies differ in the type of simulation they evoke in viewers: viewers might be prompted to simulate an unexpected death (in case of an attack or accident) or an expected death (in case of a terminal illness), for example, or they might simulate the rituals surrounding death (in case of a funeral) rather than death itself.

The above factors are relevant to analyze because they can reveal more about what viewers simulate while watching death scenes in movies. Therefore, the following research question was formulated:

RQ2 How is death portrayed in popular movies?

Answering the two research questions will help us understand how movies assign meaning to death and to what extent movies invite viewers to simulate death as it realistically occurs in real life, which, in a broader sense, advances our understanding of how eudaimonic entertainment may help people to cope with existential fears.

## Method

### Movie Set

The IMDB database was used to assemble a set of sixty popular drama and action movies released between 2012 and 2021. We searched for feature films in English with a minimum of 10,000 user votes, using the keyword ‘death’. A total of 161 movies fulfilled the criteria. For each year, six movies were included in our sample: three movies with the highest viewer ratings in the genre category “drama” and three movies with the highest viewer ratings in the genre category “action”.^1,^^2^ Our final sample consisted of 60 movies.

We chose to include two genres for generalization purposes. We chose drama movies and action movies specifically because death events occur in both these genres (cf. Schultz & Huet, 2001), which make these genres suitable for the purposes of our study. Drama and action movies can furthermore be considered as stable, basic genres (Visch, 2007) and are among the most successful movie genres (The Numbers, 2024). Including these two genres thus enables us to examine how death is portrayed in movies that are seen by large numbers of people. An overview of the movies is provided in the Appendix.

### Quantitative Analysis

All movies were analyzed in a two-step procedure. In the first step the movies were coded on three variables. First, it was determined if (at least) one of the characters dies in the movie. To that end, coders analyzed whether or not a living character died within the period of time depicted by the movie. The period of time depicted by the movie was defined as ranging from the time at which the first event occurred to the time at which the last event occurred, irrespective of the order in which these events were presented in the film. Second, it was determined if death was a central theme of the movie. If death was a recurring theme of the movie, and/or a recurring topic of conversation between characters, and if it significantly affected the storyline, it was considered a central theme. If, however, death was not a recurring theme, and/or not a recurring topic of conversation between characters, and if it did not significantly affect the storyline, it was not considered a central theme (Bridgewater et al., 2021).

Third, the main death-related event was identified. Death-related events were considered to be all events, actions, and situations relating to death (Schultz & Huet, 2001). This included actual deaths of characters in the movie, but also events that did not include an actual death, such as conversations about a specific death or death in general, threats of death, and memorials. If a movie had one death-related event, this event was qualified as the main death-related event. If a movie had more than one death-related event, it was determined which of these events was the most important death-related event. A death-related event was qualified as main death-related event if it was crucial to the integrity and development of the story and moved the story along its trajectory (cf. Chatman, 1978). If a movie turned out to have more than one main death-related event, it was decided which one was most essential, i.e., which one had the largest impact on the story. If it was not possible to decide this, the event that was shown first in the movie was selected as the main death-related event. Coders were instructed to report the starting time and ending time of the scene depicting the main death-related event and to write a short description of the event.

In the second step of the analysis, the main death-related event was further analyzed on a number of variables. Crucial for the goal of this study, the role of the main death-related event was determined in relation to both (a) the underlying narrative structure (the story as it chronologically happened) and (b) the surface structure of the movie (the order in which the events are shown) (Chatman, 1978). For the *underlying narrative structure*, it was determined if the death-related event was story-initial, story-intermediate, or story-terminating (Hagin, 2010). Story-initial deaths were defined as deaths occurring in the first quarter of the story. Story-intermediate deaths were defined as deaths occurring in the second or third quarter of the story. Story-terminating deaths were defined as deaths occurring in the final quarter of the story. For example, if a movie depicted events occurring within a period of twelve months, the main death-related event was analyzed as story-initial if it took place within the first three months, as story-intermediate if it took place between month 4 and month 8, or as story-terminating if it took place between month 9 and 12 – regardless of the order in which the events were presented to the viewer.

For the *surface structure*, we followed Field (2005) and divided each movie into four quarters and then determined if the main death-related event was shown in the beginning of the movie (i.e., in the first quarter), in the middle of the movie (i.e., in the second or third quarter), or in the end of the movie (i.e., in the fourth quarter). For example, if a movie lasted for 120 minutes, the main death-related event was analyzed as shown in the beginning if it was shown in the first 30 minutes of the movie, as shown in the middle of the movie if it was shown between minute 31 and minute 90, and as shown in the end of the movie if it was shown between minute 91 and 120 – regardless of whether the movie’s main death-related event was categorized as story-initial, story-intermediate, or story-terminating in the underlying narrative structure (see above).

The main death-related events were analyzed on a number of additional variables. First, the *type of death* event was determined. Eight types were based on the findings of Schultz and Huet (2001): (1) Funeral/burial: a death-related event involving the funeral or burial of a dead character, or a memorial service; (2) Afterlife: a death-related event depicting a character’s life after death, the existence of an afterlife, ghosts, heaven, or hell; (3) Accident: a death-related event in which a character’s life is put in danger and/or comes to an end by accident (i.e. involuntarily and unplanned), such as traffic accidents and drownings; (4) Threat/risk: a death-related event which poses a direct threat to a character’s life, which can either be caused by the character him- or herself or by someone else; (5) Attack: a type of death-related event in which a character’s is physically attacked by another character; (6) Self-harm: a type of death-related event in which a character intentionally harms themselves with the purpose of taking their own life; (7) Medical: a type of death-related event in which a character shows symptoms of, is diagnosed with, is treated for, and/or dies from a disease (e.g., cancer, AIDS); (8) Conversation/mention of death: a type of a death-related event in which (anything relating to) death or a dead person is talked about. The supplementary category of (9) Natural death was added to the list of death types to account for natural deaths that were not clearly caused by a disease. A combination of types could apply to a single death-related event. For each type of death-related event, it was therefore determined if it applied to the main death-related event or not.

Then, it was determined if the main death-related event actually *resulted in or from a character’s death*. If the event did not result in or from an actual death (for example in case it was a conversation about death in general), coding of that movie ended. If the event did result in or from an actual death, the role of the dead character was subsequently determined. This could be a main character, the loved one of a main character, an acquaintance of a main character, a stranger to the main character, or an enemy of a main character. The *gender of the dead character* was also analyzed (female, male, or other). Finally, following Graham et al. (2018), it was determined if the character’s death was *shown explicitly* (i.e., if the character’s dead, motionless body was shown) or not.

### Intercoder Reliability

Two coders familiarized themselves with the codebook and discussed any uncertainties and inconsistencies, after which the codebook was further refined. Both coders independently watched and analyzed a movie (not included in the main set of selected movies) for training purposes. After comparing and discussing their codings, some final minor adjustments were made to the codebook. For the main analysis, the first coder watched and analyzed all sixty movies. The second coder independently watched and analyzed twelve randomly selected movies. As a first step, both coders independently identified the main death-related event. For eleven out of the twelve movies, the coders agreed on the main death-related event. For the twelfth movie, the coders discussed their choices and came to agree on which event was the most important main death-related event. Upon agreeing on the main death-related event for each movie, the coders continued with step 2 of the analysis and independently analyzed the main death-related events on the remaining variables.

The intercoder reliability scores were sufficient to good for the following variables: Character dies (Krippendorff’s α = 1.00); Threat/risk (Krippendorff’s α = .807); Attack (Krippendorff’s α = 1.00); Self-harm (Krippendorff’s α = 1.00); Conversation (Krippendorff’s α = .830); Role of death in underlying structure (Krippendorff’s α = .720); Role of death in surface structure (Krippendorff’s α = .671); Event results in/from death (Krippendorff’s α = 1.00); Role of dead character (Krippendorff’s α = .880); Death explicitly shown (α = 1.00). Reliability scores were insufficient for the following variables: Death central theme (Krippendorff’s α = .000); Medical death (Krippendorff’s α = .635); Natural death (Krippendorff’s α = .000); Gender dead character (Krippendorff’s α = .632). However, the percentage agreement was high for each of these variables (>90%) and low alpha scores were caused by singular discrepancies in rare categories. We therefore decided to retain these variables in our analyses. Finally, Krippendorff’s α could not be calculated for three variables because there was no variation in the categories: Funeral; Afterlife; Accident. The percentage agreement was 100% for each of these variables.

### Qualitative Analysis

The quantitative analysis was supplemented with a qualitative analysis, in which the main death-related events were further examined by the first coder through a thematic analysis. Thematic analysis refers to a set or “family” of methods that can be used to identify and make sense of shared meanings across data items (Braun & Clarke, 2012, 2023). It involves a bottom-up process of applying codes to data items (here: death-related events in movies) and subsequently clustering these codes in order to identify themes that represent “some level of *patterned* response or meaning within the data set” (Braun & Clarke, 2006, p. 82).

We specifically conducted a reflexive thematic analysis in a deductive way (Braun & Clarke, 2021), meaning that the movies were analyzed through the lens of Hagin’s (2010) framework (a character’s death can be meaningful in relation to the past, present, or both) while maintaining flexibility in interpreting the precise nature and aspects of this meaning. The advantage of using thematic analysis was that its flexibility allowed us to keep the unit of analysis identical to the unit of analysis in the quantitative analysis (i.e., the main death-related event) and, hence, relate the findings to the results of the quantitative analysis, but that we could also analyze the death-related events in relation to the entire movies and, hence, add additional depth to the findings of the quantitative analysis.

Following the procedure for reflexive thematic analysis (Braun & Clarke, 2006), all main death-related events were first watched closely multiple times. In the second step of the analysis, the events received initial codes that purely described the events (e.g., “end of relationship”, “revenge”). In the third step, the initial codes were then clustered into themes (e.g., “end of relationship” was clustered with similar codes into the larger theme of “separation”, “revenge” was clustered with similar codes into the larger theme of “salvation”). These themes were in step four reviewed and checked against the initial codes as well as the complete set of main death-related events, after which the themes were refined and received a definitive name in step five, and were reported in the sixth and final step.

## Results

The results in this section are based on the combined findings for drama movies and action movies. We also performed contrastive analyses to identify potential differences between these genres. For most of the variables, there were no differences between drama movies and action movies. In those cases where we did find differences, these are reported in footnotes.

### Quantitative Analysis

#### Character Deaths

In 95% of the movies, at least one of the characters died. Death was a central theme in 80% of all movies.

#### Role of Death

**Figure 1.**
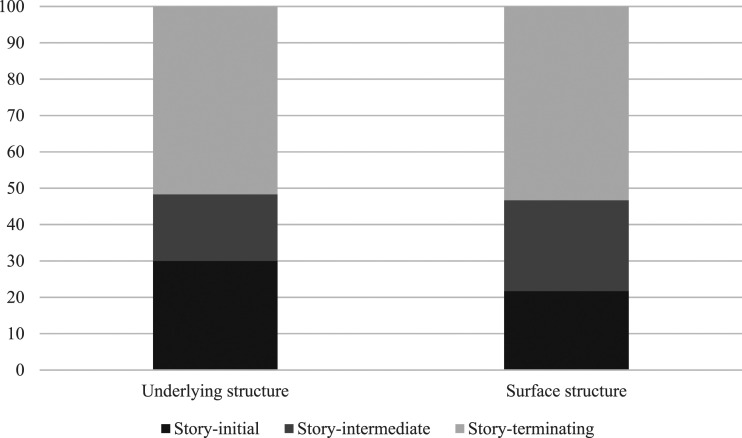
Role of death in the narrative structure of movies.

The role of death was analyzed in two ways: first, by analyzing the role of death in the underlying narrative structure of the movie, and second, by analyzing the occurrence of death in the surface structure of the movie. Figure 1 shows the percentages of movies in which the role of death was story-initial, story-intermediate, or story-terminating in the underlying narrative structure and in the surface structure.

Figure 1 shows that death-related events tend to function as a story’s ending rather than a story’s beginning or a story’s middle, both in the underlying narrative structure and in the surface structure. In 52 movies (86.7%), the role of death in the surface structure of the movie was equal to the role of death in the underlying narrative structure. This result indicates that, for movies in which death occurs at and is shown at the end, death is depicted as an ending in a quite literal way: it ends the journey of the characters (in the narrative structure) as well as the viewing journey of the audience (in the surface structure).

In the remaining 8 movies (5 drama, 3 action)^3^, there was a difference between the surface structure and the underlying structure. In six of these, the role of death in the underlying narrative structure was story-initial but the death-related event was not shown in the beginning of the movie but either in the middle (five movies) or at the end (one movie). In one movie the role of death was story-intermediate but the death was shown only at the end of the movie. The effect of these compositions, in which death is shown later than it actually occurred, is that viewers are asked to reconsider the current life, views and actions of a character in light of a death-related event from the past. The meaning of death is thus that it *changes* our view on the meaning of life. The reversed occurred in one movie, in which the role of death was story-terminating but the death was shown in the beginning of the movie. The effect of this composition, in which death is shown before it actually occurs, is that viewers are invited to ascribe meaning to all events and situations in light of a looming death. Here, the meaning of death is that it *adds* meaning to life.

#### Type of Death

Table 1 below shows which type of events applied to the main death-related events.

**Table 1.** Type of Main Death-Related Events.

Type	Occurrence
Attack	32 (53.3%)
Conversation	22 (36.7%)
Threat/risk	14 (23.3%)
Self-harm	6 (10%)
Medical	6 (10%)
Accident	3 (5%)
Natural death	3 (5%)
Funeral	0 (0%)
Afterlife	0 (0%)

*Note.* More than one category can apply to a single death-related event.

Table 1 shows that the majority of main death-related events were attacks, followed by conversations about death and threats or risks. Notably, there were no death-related events involving funerals or afterlife. Medical deaths, accidents, natural deaths, and self-harm events were rare.^4^

#### Character Characteristics

The majority of the dead characters was male (72.9%). In the movies in which the main death-related event resulted in the actual death of a character (*N* = 48), it was most often a main character or a main character’s loved one who passed away; and deaths of main characters and deaths of loved ones occurred equally frequently. Table 2 shows an overview of the roles of the dead characters.

**Table 2.** Role of the Dead Character.

Dead character	Occurrence
Main character	14 (29.2%)
Loved one	14 (29.2%)
Enemy	9 (18.8%)
Stranger	6 (12.5%)
Acquaintance	5 (10.4%)

#### Portrayal

In most of the movies in which the main death-related event involved the actual death of a character, that death was shown explicitly (31 out of 48 movies; 64.6%).

### Qualitative Analysis

The qualitative analysis revealed several themes, which largely mapped onto the role of death in the underlying narrative structure. Results of the qualitative analysis will therefore be discussed in relation to the role of death in the narrative structure of movies.

#### Story-Initial Deaths: Growth and Avoidance

A closer look at movies in which death was story-initial reveals different ways in which death influences characters’ lives. In several movies, death puts a character in a new position (cf. Hagin, 2010). In *Spider-Man: Into the Spider-Verse*, the death of Spider-man forces Miles to become the new Spider-Man. Similarly, in *Deadpool II*, the wife of main character Wade dies at the beginning of the movie. The loss of his wife puts Wade in a deep depression, until he joins the X-Men as a form of healing. Throughout the movie, Wade has several visions of his wife which guide his actions and awaken his ability to care for others. These movies relate death to the theme of *growth* and show that death becomes meaningful through its impact on the lives of others by stimulating (or forcing) them to grow.

In other movies, story-initial deaths make characters leave their past behind in an attempt to start a new life. This is for example the case in *Manchester by the Sea*. This movie tells the story of Lee, a depressed man who feels responsible for the death of his three children. They died in a fire after a drunken Lee forgot to put a guard in front of the fireplace before he left the house to buy more alcohol. Lee and his wife divorced after the incident and Lee left their hometown Manchester-by-the-Sea. When Lee’s brother dies, Lee is forced to return to Manchester-by-the-Sea because he is named as the guardian of his brother’s son. Lee is thus forced to face his past – his ex-wife, the people who know him and his past and their judgments – in order to move forward. In *Shang-Chi and the Legend of the Ten Rings*, the mother of main character Shang-Chi is murdered when he is eleven years old. His father starts training him in martial arts and forces him to kill the murderer of his mother. Shang-Chi is traumatized by these events and flees to San Francisco, where he attempts to start a new life. One day he is robbed by people from his past and forced to return home to fight his father, before he can resume his life in San Francisco. Both of these movies relate death to the theme of *avoidance*, and show that death avoidance is essentially ineffective: death and grief must be faced in order to move forward. In this way, these movies relate death to the theme of *growth* as well: overcoming death avoidance and facing grief results in personal growth.

#### Story-Intermediate Deaths: Unification

Story-intermediate death events were least common in the movie set and these events involved least frequently an actual death (63.6%). For example, in the biographical war movie *Hacksaw Ridge*, the main death-event is a conversation between main character Desmond and his squad mate Smitty during the Battle of Okinawa. Desmond tells Smitty how he once almost shot his abusive father, who was pointing a gun at Desmond’s mother. The incident caused Desmond to develop a great aversion to guns. Desmond’s fellow soldiers had, up until then, been treating him as an outsider because he refused to carry a rifle, but this conversation about a death-related event from Desmond’s past creates mutual understanding and marks the beginning of a strong bond between Desmond and Smitty. The conversation reveals the truth about the past (i.e., why Desmond has been refusing to use weapons) but is also meaningful in relation to the future: his refusal to carry a rifle will no longer be seen as cowardly, but as courageous, in particular when he will later save dozens of wounded soldiers.

In *Star Wars: Rogue One*, the story-intermediate death event does result in an actual death. In her early childhood, Jyn is separated from her father, who is taken away and forced to build an extremely deadly weapon for the Galactic Empire. Fifteen years later, Jyn receives a message from her father. She goes out to find him but he becomes severely injured in the meantime. Jyn eventually finds her father and they reunite, just moments before he passes away in her arms. A theme that emerges from these two movies is that death *unites* people.

#### Story-Terminating Deaths: Salvation and Separation

Several movies with a story-terminating death can be seen as prototypical examples of a death that puts an end to evil (Hagin, 2010). In the drama movie *Zero Dark Thirty*, CIA analyst Mia is determined to hunt down Osama bin Laden. After searching him for years and risking her own life multiple times, she finally manages to track him down, which ultimately leads to his death and puts an end to his evil actions. Likewise, in the drama movie *Prisoners*, Mrs. Jones is killed right before she wants to kill the little girl Anna. Similar story-terminating deaths are found in a number of action movies, for example: in *Gentlemen*, Mickey saves his wife by killing Dry Eyes; in *Iron Man 3*, Iron Man’s wife saves Iron Man by killing Killian Aldrich; and near the end of *Mad Max: Fury Road*, Furiosa manages to kill Joe, thereby making an end to his despotic rule over the land. All of these movies relate death to the theme of *salvation*. In some movies, *salvation* was achieved through self-sacrifice. In *Fast & Furious 6*, for example, Gisele sacrifices herself to save Han, who was being attacked. Similarly, in *Guardians of the Galaxy Vol. 2*, Yondu sacrifices himself to save Peter by giving Peter his spacesuit, after which he – instead of Peter – freezes up in space. A final type of *salvation* was found in the drama movie *A Star is Born*, when Jack commits suicide to free himself and his girlfriend Ally from his substance abuse that ruined his career and had started to ruin Ally’s career.

A second theme that emerged from the analysis of story-terminating deaths was that of *separation*. In the movie *West Side Story*, for example, Tony’s death separates him from Maria, the woman he loves. Tony’s death abruptly ends their blossoming love. At the end of *I, Daniel Blake*, main character Daniel dies from a heart attack. His death separates him from his friend Katie and her daughter Daisy, who had grown close to Daniel. Similarly, at the end of *Me and Earl and the Dying Girl*, 17-year-old Rachel dies from leukemia. Her death separates her from her friend Greg, the movie’s main character, who had been an important support to her during her chemotherapy treatment. Although death results in separation in these movies, it can be seen as meaningful in the sense that it underscores the value of interpersonal relationships.

## Conclusion and Discussion

Grounded in research in the domains of media psychology, communication studies, and death studies, this study combined the concepts of eudaimonic entertainment, narrative structure and narrative simulation to examine what popular movies communicate to the audience about the meaning of death so as to increase our understanding of whether and how these movies might help people to cope with death and death anxiety. Research question 1 asked how death is related to the narrative structure of movies. Results of the quantitative analysis show that death events tend to be story-terminating. This implies that, for most movies (drama as well as action), death is depicted as meaningful in relation to the past (Hagin, 2010): it retrospectively adds meaning to the lives and journeys of people. It also means that death is seen as the final station, whereas in real life, it rarely is for the ones who are left behind. This study shows that movies tend to end with a traumatizing event, but such traumas can also be the starting point of new journeys, deeper interpersonal connections, and personal growth (Tedeschi & Calhoun, 1996). The latter view of death was expressed in a smaller number of movies, in which death – most often the death of a loved one – was story-initial and therefore meaningful in relation to the future of the main character (Hagin, 2010).

Results of the qualitative thematic analysis of death events add important nuances to the above findings. Although a number of movies related story-terminating deaths to a theme of separation, other movies ascribed alternative meanings to such deaths by depicting it as an event that can lead to salvation. Furthermore, story-intermediate deaths may lead to unification and story-initial deaths may lead to personal growth. Several movies related a story-initial death to the theme of avoidance and showed not only that death avoidance, which is a highly common attitude towards death (e.g., Furer & Walker, 2008), is an ineffective coping mechanism but also that death avoidance can be overcome. This finding is in line with the observation that death acceptance is a common theme in movies, realized through the portrayal of characters acknowledging or even embracing the inevitability of death (Niemiec & Schulenberg, 2011). Hence, although death often marks the ending of a movie and the ending of the story it tells, it is not by definition depicted as meaningless; instead, movies display a diverse range of ways in which death can be meaningful to the lives of individuals.

Research question 2 asked how death is depicted in popular movies, in order to advance our understanding of what viewers precisely simulate while watching death scenes. The results showed, first of all, that main characters and their loved ones are the most likely characters to die, in drama movies as well as action movies. This finding is in contrast with Gibson’s (2001) observation that the hero in an action movie never dies. Moreover, it implies that viewers simulate death in different ways: either their own death (when the main character dies) or the confrontation with the death of a loved one and the subsequent feelings of loss and grief (when the main character’s loved one dies).

Second, the majority of the movies included explicit death portrayals, that is: depictions of dead bodies. While being absorbed into the story and identifying with its characters, viewers thus closely approach and simulate death. At the same time, viewers know that the story world is fictitious and that they can escape from it at any time (Gibson, 2001). As such, explicit death portrayals can be argued to provide social scripts for dying (Knox, 2006) within the safe context of a fictitious world. Note, however, that the portrayal of death might be different for other types of movies: whereas the current study found that about 65% of the movies included an explicit death portrayal, an analysis of recent Disney and Pixar movies showed that in only 20% of these movies death is shown explicitly (Graham et al., 2018).

The finding that most movies show death explicitly contradicts, at first sight, the contention that death tends to be hidden in modern Western societies, and thus the public denial of death (Ariès, 1975). However, explicit death scenes can be understood as exemplifications of what Jacobsen (2016) calls ‘the spectacular death’. In Jacobsen’s (2016) view, we currently live in a post-denial age in which death is not hidden, forbidden or a taboo; instead, death is more than ever all around us, in particular in media representations, and we have become spectators of death in the sense that we witness and approach death from a safe distance – via television, computer and phone screens.

In contrast with the abundant presence of death in the media, we still rarely encounter death in real life because it is typically handled in professional rather than private spaces (Jacobsen, 2016). The representation of death in the media is therefore crucial to our understanding of death and feelings towards death (e.g., Clarke, 2006), but results of the current study showed, thirdly, that movies tend to paint an unrealistic view of *how* people die: more than half of all deaths were attacks, whereas in reality most people die from medical issues or accidents rather than violence (CDC, 2021). Medical deaths, accidents, and natural deaths were rare in our analysis, however. This contrast exemplifies the “widening gap and experiential differential between media/technological death culture and ‘real life’ contexts and temporalities of death and bereavement” (Gibson, 2007, p. 415).

Moreover, Schultz and Huet (2001) reported similar findings for movies released between 1980 and 1994, which points towards a persistent tendency among movie makers to associate death with violence. Decades of research on cultivation theory (Gerbner & Gross, 1976) have demonstrated that the overrepresentation of violence in the media is positively correlated with fear and perceived danger in viewers (see, e.g., Shanahan & Morgan, 1999). Consequently, the rather uniform and violent “movie way of death” might reinforce rather than reduce people’s fear of death. Indeed, studies showing the potential of movies to ease viewers’ fear of death used stimulus materials in which characters died in more realistic, i.e., medical contexts (Das et al., 2019; Rieger & Hofer, 2017), which could mean that the positive effects of movies on viewers’ existential fears might be limited to a specific, relatively small group of movies.

The films included in this analysis were popular movies that are seen by millions of people, which enables us to draw conclusions about what large audiences learn about death from watching movies. A limitation of this approach is that other genres and movie types, such as arthouse movies, were not included while death may be portrayed differently in such movies. Another limitation is that our analysis focused on one death event per movie, the most significant event, while many movies included multiple death events. The way in which death is portrayed in a single movie may thus be more diverse than we could determine in this study. Likewise, viewers may simulate death in various contexts during a single movie and ultimately develop a rich and multifaceted understanding of the meaning of death. Future research could help to arrive at a more comprehensive and inclusive view on the role and meaning of death in moves and, in addition, to further unravel the causal relations between death depictions in movies and viewers’ simulation, understanding, and fear of death.

This study adopted a novel approach by examining not only the number of deaths in movies but also, and more prominently, the role and meaning of death in popular movies. The process of meaning-making and is central to how people cope with personal loss (Gillies & Neimeyer, 2006). Results of our study thus provide more insight into the ways in which movies may help people to deal with death in real life: by watching movies about death, they simulate a process of meaning-making which may facilitate this process in real life. Specifically, although we found that movies tend to depict death as an ending, and thereby as meaningful in relation to the past (Hagin, 2010), results of our study showed that death is also shown as meaningful in the sense that it can lead to growth, unification and salvation. Viewers are furthermore invited to closely approach death through explicit death portrayals, and to simulate both their own mortality and the mortality of their loved ones, although the way in which movie characters die tends to be unrealistic. These findings contribute to our understanding of how movies may and may not help viewers to cope with existential fears and, more generally, to knowledge about the eudaimonic aspects of entertainment. As such, they underline why studying death in movies is relevant for research in the fields of death studies as well as media psychology and communication studies.

## Notes

1. Viewer ratings on IMDB are provided on a scale from 1 (low) to 10 (high).

2. Movies with both “drama” and “action” as genre labels were excluded so that we were able to also make a clean comparison between drama and action movies.

3. Drama: *The Imitation Game*, *Manchester by the Sea*, *Knives Out*, *The Irishman*, *A Quiet Place Part II*. Action: *Extraction*, *Shang-Chi and the Legend of the Ten Rings*, *Captain America: Civil War*.

4. Comparing the drama movies with the action movies, it was found that attacks were more frequent in action movies (χ^2^(1) = 6.70, *p* = .010) whereas self-harm events were more frequent in drama movies (χ^2^(1) = 6.67, *p* = .010). The other type of death-related events occurred equally frequent in drama movies and action movies (all *p*’s > .076).

5. As of February 2022.

## Appendix

**Table:**

Year	Drama	Rating^5^	Action	Rating
2012	Django Unchained	8.4	The Avengers	8.0
	The Impossible	7.6	Skyfall	7.8
	Zero Dark Thirty	7.4	End of Watch	7.6
2013	Prisoners	8.1	Star Trek: Into darkness	7.7
	Dallas Buyers Club	8.0	Iron Man 3	7.1
	Short term 12	8.0	Fast & Furious 6	7.0
2014	Interstellar	8.6	Guardians of the Galaxy	8.0
	Gone Girl	8.1	Edge of Tomorrow	7.9
	The Imitation Game	8.0	X-Men: Days of future past	7.9
2015	Straight outta Compton	7.8	Mad Max: Fury Road	8.1
	The Hateful Eight	7.8	Star Wars Episode VII: The Force Awakens	7.9
	Me and Earl and the Dying Girl	7.7	Mission: Impossible: Rogue Nation	7.4
2016	Hacksaw ridge	8.1	Deadpool	8.0
	I, Daniel Blake	7.9	Star Wars: Rogue One	7.8
	Manchester by the Sea	7.8	Captain America: Civil War	7.8
2017	Ayla: The Daughter of War	8.4	Thor: Ragnarok	7.9
	Three Billboards outside Ebbing, Missouri	8.1	Guardians of the Galaxy Vol. 2	7.6
	Wind River	7.7	John Wick: Chapter 2	7.5
2018	Searching	7.6	Spider-Man: Into the Spider-Verse	8.4
	A Star is Born	7.6		
	Blindspotting	7.4	Avengers: Infinity War	8.4
			Deadpool 2	7.7
2019	Joker	8.4	The Gentlemen	7.8
	Knives out	7.9	John Wick: Chapter 3: Parabellum	7.4
	The Irishman	7.8	Alita: Battle Angel	7.3
2020	Nomadland	7.3	Tenet	7.4
	A Quiet Place part II	7.3	Monster Problems (Love and Monsters)	7.0
	Pieces of a Woman	7.0	Extraction	6.7
2021	West Side Story	7.8	Spider-Man: No way home	8.7
	Judas and the Black Messiah	7.5	Zack Snyder’s Justice League	8.1
	The French Dispatch	7.3	Shang-Chi and the Legend of the Ten Rings	7.5
